# Validation of the Scottish Inflammatory Prognostic Score (SIPS) in NSCLC Patients Treated with First-Line Pembrolizumab

**DOI:** 10.3390/cancers16223833

**Published:** 2024-11-14

**Authors:** Igor Gomez-Randulfe, Fabio Gomes, Melanie MacKean, Iain Phillips, Mark Stares

**Affiliations:** 1 The Christie NHS Foundation Trust, Manchester M20 4BX, UK; igor.gomezrandulfe@nhs.net (I.G.-R.);; 2 Edinburgh Cancer Centre, NHS Lothian, Edinburgh EH4 2XU, UK; 3 Cancer Research UK Scotland Centre (Edinburgh), University of Edinburgh, Edinburgh EH4 2XR, UK

**Keywords:** non-small cell lung cancer (NSCLC), Scottish Inflammatory Prognostic Score (SIPS), prognostic biomarker, systemic inflammation, programmed death ligand 1 (PD-L1), pembrolizumab

## Abstract

Background: The Scottish Inflammatory Prognostic Score (SIPS), combining albumin (≥/<35 g/L) and neutrophil count (≤/>7.5 × 10^9^/L), has been identified as a prognostic biomarker for patients with non-small cell lung cancer (NSCLC) undergoing treatment with pembrolizumab monotherapy. We sought to validate this biomarker of systemic inflammation in an external cohort. Methods: Patients treated with first-line pembrolizumab for advanced NSCLC with programmed death-ligand 1 (PD-L1) expression ≥ 50% at an English cancer centre were identified. Pre-treatment clinicopathological characteristics and the SIPS were recorded. The relationship between these and progression-free survival (PFS) and overall survival (OS) was examined. Results: Among 257 patients evaluated, 56% (*n* = 144) were classified as SIPS 0, 36% (*n* = 93) as SIPS 1, and 8% (*n* = 20) as SIPS 2. Factors such as age, performance status (PS) and brain metastases presence were significantly correlated with SIPS categories. Multivariate analysis revealed that both SIPS and PD-L1 status were independently associated with PFS and OS. The combination of SIPS with either PS or PD-L1 expression enhanced the ability to detect patients with the most favourable or poorest survival. Conclusions: Our study confirms the prognostic significance of the SIPS in patients with advanced NSCLC treated with pembrolizumab in the context of high PD-L1 expression. SIPS offers a straightforward, clinically applicable approach to patient stratification, potentially guiding therapeutic decisions and enhancing outcomes in advanced NSCLC. Future research should focus on validating these findings in prospective studies and exploring the integration of SIPS into clinical practice, alongside other prognostic markers, to optimize treatment strategies.

## Simple Summary

In this study we examined whether a specific blood test score, the Scottish Inflammatory Prognostic Score (SIPS), could help predict outcomes in people with advanced lung cancer receiving immunotherapy. Immunotherapy, which uses the body’s immune system to fight cancer, is effective for some patients but not all. SIPS is based on two common blood measures: albumin (a protein) and neutrophils (a type of immune cell). Our findings suggest that patients with low SIPS scores, indicating lower inflammation, generally lived longer and had slower cancer progression. SIPS could become a helpful tool for doctors to decide on treatments and guide patients about their prognosis. Using SIPS alongside other health information may improve care by helping select the most suitable therapies for people with advanced lung cancer. Further studies could confirm these findings and support SIPS as a standard measure in clinical care.

## 1. Introduction

Advanced non-small cell lung cancer (aNSCLC) represents 85% of new lung cancer diagnoses, being one of the most frequent causes of mortality due to cancer in the world [1]. Over the past decade, the treatment landscape for aNSCLC has dramatically shifted with the introduction of immune checkpoint inhibitors (ICIs). Pembrolizumab, an anti-programmed cell death protein 1 (PD-1) agent, has proven its superiority over cytotoxic chemotherapy in patients whose tumours exhibit high programmed death-ligand 1 (PD-L1) expression (>50%) [2]. This benefit was also evident in a trial comparing pembrolizumab to chemotherapy in patients diagnosed with aNSCLC and PD-L1 expression above 1%, although the gain was mainly driven by tumours with high PD-L1 levels [3]. Complicating the landscape, pembrolizumab combined with chemotherapy (i.e., chemoimmunotherapy) has demonstrated superior outcomes irrespective of PD-L1 expression in both squamous and non-squamous NSCLC populations [4,5].

Currently, no randomized prospective trials have compared immunotherapy alone with chemoimmunotherapy in patients with PD-L1 expression ≥ 50%. Pembrolizumab monotherapy remains the predominant choice in this context in the United Kingdom, with chemoimmunotherapy used at the treating clinician’s discretion, typically being reserved for cases with extensive tumour burden. Unfortunately, even though it results in a doubling of the 5-year overall survival (OS) rate compared to chemotherapy (31.9% versus 16.3%, respectively), not all eligible patients will respond to pembrolizumab [2]. The objective response rates were only 45%, with approximately 20% of patients succumbing within 6 months of initiating treatment [2]. Prognostic and/or predictive biomarkers that can be easily implemented in clinical practice could help identify patients with a poor prognosis and eventually guide treatment selection.

Tumour-driven inflammation is one of the hallmarks of cancer, and it has been shown to play a pro-tumoral role [6]. Systemic inflammation indices can be derived from standard peripheral blood metrics like blood count, albumin levels, or lactate dehydrogenase (LDH) [7]. Prior to the immunotherapy era, scores assessing systemic inflammation were found to possess prognostic significance across various tumours [8]. In recent years, numerous scores utilizing routine blood parameters have been proposed [9]. Some of them include peripheral neutrophil and lymphocyte count, either alone or as part of composites scores like neutrophil to lymphocyte ratio (NLR), derived neutrophil to lymphocyte ratio (dNLR), or platelet to lymphocyte ratio (PLR) [10,11]. More recently, some scores have been published considering the value of LDH in addition to the peripheral blood count [7,12]. Unfortunately, these scores have not been widely implemented due to a variety of reasons, among them the lack of external validation. It has been reported that only 5% of predictive models studies report external validation in medicine [13].

Recently, Stares et al. published a novel score that reflects systemic inflammation using easy-to-access data in everyday clinic, the Scottish Inflammatory Prognostic Score (SIPS) [14]. It allocates 1 point for baseline albumin < 35 g/L or neutrophil count ≥ 7.5 × 10^9^/L, resulting in a three-tier categorical score. It was found to predict and stratify progression-free survival (PFS) and overall survival (OS) in patients diagnosed with aNSCLC and PD-L1 expression ≥ 50% undergoing first-line pembrolizumab treatment in a real-world setting [14]. This study aims to validate the prognostic utility of the SIPS in an independent retrospective cohort of patients with high PD-L1 expression receiving pembrolizumab as the first-line therapy for NSCLC.

## 2. Methods

### 2.1. Study Population

All consecutive patients diagnosed with locally advanced or metastatic NSCLC who exhibited a PD-L1 expression of 50% or greater received first-line pembrolizumab, and who initiated their first cycle between June 2017 and May 2023 at The Christie NHS Foundation Trust, UK, were considered eligible. PD-L1 tumour proportion scores were tested by immunohistochemistry (Ventana 263 clone, Ventana Medical Systems, Tucson, AZ, USA). Patients did not have an EGFR, ALK, or ROS1 actionable genomic alteration. Clinical and demographic data were sourced from patient’s electronic health records. We gathered information on age, sex, histology, PD-L1 status, Kirsten rat sarcoma viral oncogene homolog (KRAS) mutational status, Eastern Cooperative Oncology Group performance status (ECOG PS), and the presence of baseline brain metastases. Laboratory data, including neutrophil count (NC) and albumin, were obtained from routine blood samples taken within a 14-day window prior to treatment. PD-L1 expression was dichotomized into <90% and ≥90% based on previous studies that have shown longer PFS and OS in the latter group [15,16].

SIPS was determined by allocating 1 point each for baseline albumin < 35 g/L and neutrophil count > 7.5 × 10^9^/L. This resulted in a three-tier classification: 0 for favourable prognosis, 1 for intermediate prognosis, and 2 for poor prognosis. Radiological evaluations were conducted according to the investigator’s discretion, utilizing Response Evaluation Criteria in Solid Tumours (RECIST) 1.1 criteria.

### 2.2. Statistical Analysis

PFS was defined as the time elapsed between the first dose of pembrolizumab and the date of radiological progressive disease, death, or date of last follow-up if censored. OS was calculated from the date of the first dose of pembrolizumab to the date of death or censorship. Survival curves were plotted using Kaplan–Meier methods and the log-rank test was applied. Survival analysis was carried out using Cox’s proportional hazards model, and hazard ratios (HRs) were calculated. Multivariate survival analysis was performed using a stepwise backward procedure to derive a final model of the variables that had a significant independent relationship with survival. To remove a variable from the model, the corresponding *p*-value had to be >0.10. All analyses were carried out in SPSS Version 24.0 (SPSS Inc., Portsmouth, Hampshire, UK). This study adheres to the Reporting Recommendations for Tumour Marker Prognostic Studies guideline.

## 3. Results

Comprehensive baseline clinical and demographic details are outlined in Table 1. We included a total of 257 patients with a median age of 70 years (range 45–87), and 124 were females (48%). In terms of performance status, the majority were ECOG 1 (*n* = 191, 74%), followed by ECOG 0 (*n* = 36, 14%) and ECOG 2 (*n* = 30, 12%). Baseline brain metastases were identified in 32 patients (12%). Given the time period covered by this retrospective cohort, KRAS mutation status was available for only 69 patients with non-squamous histology (27%), with 37 (54%) of them having a KRAS mutation.

The majority of patients showed no evidence of raised inflammation, defined as SIPS 0 (*n* = 144, 56%). Only 8% (*n* = 20) of patients were SIPS 2. Significantly, age, PS, and the presence of brain metastases were associated with SIPS. When age was segmented into three categories (<65 years, 65–74 years, and >74 years), patients with SIPS 1 were generally younger than those with SIPS 0 or 2. PS declined patients were found to be more inflamed as determined by SIPS. Regarding brain metastases, they were more prevalent in patients with SIPS 1 compared to SIPS 0 or 2.

At the time of data cutoff, 203 (79%) patients had experienced either disease progression or death. The median PFS in the whole population was 8.9 (IQR 3.8–30.9) months. Median and minimum follow-up in censored patients was 34.2 (IQR 23.8–50.1) months and 7.1 months, respectively. Regarding OS, 181 (70%) patients had experienced death and median OS was 18.6 (IQR 6.4–43.5) months. Median and minimum follow-up in those patients who remained alive at censorship was 34.0 (IQR 2.0–49.6) months and 7.1 months, respectively.

In the univariate analysis, PD-L1 (*p* = 0.008), NC (*p* = 0.001), albumin (*p* = 0.005), and SIPS (*p* < 0.001) were predictive of PFS (Table 2). The multivariate analysis confirmed that PD-L1 (*p* = 0.006) and SIPS (*p* < 0.001) remained significantly linked to PFS.

For OS, the univariate analysis associated PS (*p* = 0.027), PD-L1 (*p* = 0.008), NC (*p* < 0.001), albumin (*p* = 0.002), and SIPS (*p* < 0.001) (Table 2). However, the multivariate analysis indicated that only PD-L1 (*p* = 0.004) and SIPS (*p* < 0.001) were significant predictors of OS. In the small number of patients for which it was available, KRAS status was not predictive of PFS (HR 0.77 (95%CI 0.44–1.37), *p* = 0.382) or OS (HR 0.71 (95%CI 0.67–1.67), *p* = 0.310).

**Figure 1:**
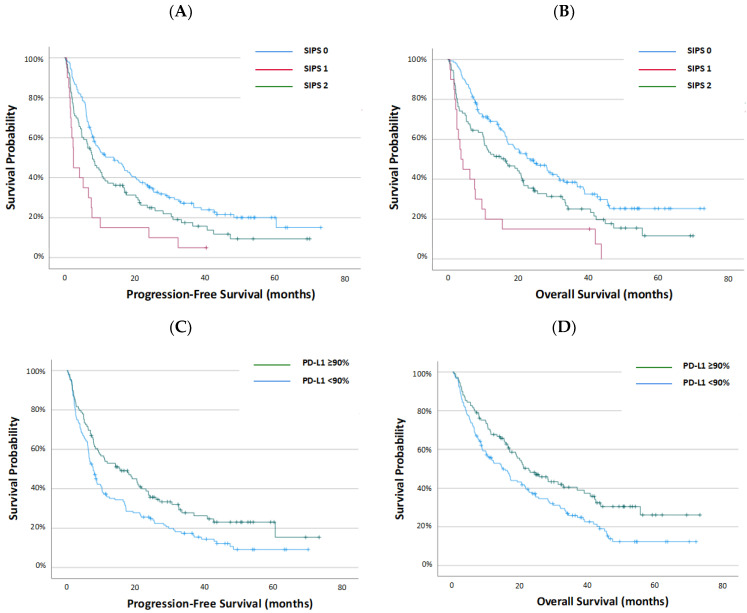
Kaplan–Meier survival curves examining the association between the Scottish Inflammatory Prognostic Score (SIPS) and (**A**) progression-free survival, and (**B**) overall survival, and PD-L1 expression and (**C**) progression-free survival, and (**D**) overall survival, in patients with non-small-cell lung cancer treated with first-line pembrolizumab monotherapy.

SIPS effectively stratified PFS: 14.0 (IQR 6.0–40.0) months for SIPS 0, 7.9 (IQR 2.6–23.6) months for SIPS 1, and a mere 2.5 (IQR 1.6–7.6) months for SIPS 2 (*p* < 0.001) (Figure 1A, Table 3). When compared to SIPS 0 patients, those with SIPS 1 had a HR of 1.43 (95% CI: 1.06–1.92) (*p* = 0.018), and SIPS 2 patients had a HR of 1.65 (95% CI: 1.29–2.11) (*p* < 0.001).

Similarly, SIPS stratified OS to 23.4 (IQR 8.4–not reached) months for SIPS 0, 16.4 (IQR 3.3–40.7) months for SIPS 1, and 3.7 (IQR 2.2–9.7) months for SIPS 2 (Figure 1B, Table 3). Relative to SIPS 0 patients, those with SIPS 1 had a HR of 1.50 (95% CI: 1.10–2.05) (*p* = 0.010), and SIPS 2 patients had a HR of 1.82 (95% CI: 1.41–2.33) (*p* < 0.001).

As described, PD-L1 expression was predictive of survival in all patients, stratifying PFS from 7.6 (IQR 2.8–24.1) months (PD-L1 < 90%) to 15.5 (5.0–40.7) months (PD-L11 ≥ 90%) (*p* = 0.007) and OS from 14.7 (IQR 5.1–36.9) months (PD-L1 < 90%) to 22.5 (9.7–not reached) months (PD-L1 ≥ 90%) (*p* = 0.007) (Figure 1C,D, Table 3).

SIPS was predictive of both PFS and OS regardless of PD-L1 expression (Table 4). The combination of PD-L1 expression ≥ 90% and SIPS0 identified a subgroup comprising 23% (*n* = 60) of all patients with the most favourable PFS (18.9 months) and OS (28.2 months). Further, 73% (*n* = 44) of these patients were alive at 12 months. No statistically significant differences in PFS or OS for patients with intermediate or high systemic inflammation with respect to PD-L1 status were identified. Shah et al. recently demonstrated that PD-L1 expression was predictive of survival in patients with non-squamous histology, but not those with squamous histology [16,17]. We therefore performed a subgroup analysis. In patients with non-squamous, but not squamous histology PD-L1 expression was predictive of PFS (HR 1.64 (95%CI 1.16–2.30), *p* = 0.005 and HR 1.07 (95%CI 0.64–1.78), *p* = 0.178, respectively) and OS (HR 1.75 (95%CI 1.20–2.55), *p* = 0.003 and HR 0.97 (95%CI 0.57–1.63), *p* = 0.965, respectively). Again, the combination of PD-L1 expression ≥ 90% and SIPS0 identified a subgroup of patients with the most favourable PFS (26.9 months) and OS (not reached after 27.6 months median follow-up).

## 4. Discussion

This study provides an external validation of the SIPS in aNSCLC patients with high PD-L1 expression who were treated with pembrolizumab. SIPS was predictive of both PFS and OS, with higher levels of inflammation associated with poorer outcomes. Our findings support the use of SIPS as a prognostic biomarker and a useful tool to help clinicians and patients when making decisions about treatment in this setting.

This is one of the few studies to externally validate a predictive model in patients with cancer. External validation is a necessary step to determine a model’s reproducibility and understand how it may be generalized across different populations. We observe several differences between our cohort and that reported in the original study by Stares et al. Our cohort contained fewer patients with PS2 (12% vs. 20%), reflecting differences in the fitness of the respective populations from which the centres draw their referrals. Of note, compared to England, Scotland has a higher lung cancer rate and a higher population-level exposure to modifiable cancer risk factors such as smoking [18,19]. This disparity may explain the more favourable OS observed in our cohort (18.6 months vs. 12.1 months), with PS, a well-recognized prognostic factor, confirmed in the univariate analysis here. However, the longer median follow-up of our cohort (34.2 months vs. 20.0 months) may also have contributed to this finding, particularly as only a small difference in PFS was seen between each cohort (8.9 months vs. 7.6 months).

The distribution of SIPS in our cohort differed significantly from that described by Stares et al. In their cohort, there was an even distribution of patients across the three SIPS categories. In contrast, our sample predominantly consisted of patients with SIPS 0 (144 patients, 56%), followed by SIPS 1 (93 patients, 36%) and SIPS 2 (20 patients, 8%). This discrepancy is reflected in the differences in fitness between the two populations. Indeed, we found that patients with a poorer PS were more likely to be highly inflamed (*p* = 0.029). Interestingly, no patients with PS 2 were SIPS 0. The association between PS and systemic inflammatory status has been described before. Dolan et al. found that the modified Glasgow Prognostic Score (mGPS: comprised albumin and c-Reactive Protein (CRP)) associated with ECOG-PS, demonstrating that the ECOG/mGPS framework remained independently associated with OS in patients with advanced cancer [20].

Despite the observed differences in population characteristics, the SIPS maintained its prognostic significance in our study. Interestingly, landmark survival rates at 3-, 6- and 12 months were similar between the 2 studies. Stares et al. commented that, in their cohort, amongst patients with SIPS 2, fewer than half were alive at 3 months. These findings are replicated in our study. This lends considerable weight to the suggestion that these patients would benefit most from discussions not to pursue treatment, or that in those that do, early clinical or radiological assessment be carried to allow earlier identification of treatment failure. Significantly, with longer median, but not minimum, follow-up, our data suggest that patients with lower levels of systemic inflammation were more likely to experience durable control, defined as PFS > 2 years: SIPS 0–32%, SIPS 1–20%, SIPS 2–15% (*p* = 0.070), or OS > 2 years: SIPS 0–42%, SIPS 1–30%, SIPS 2–15% (*p* = 0.028).

Our study benefited from the inclusion of other prognostic factors, including the presence/absence of brain metastases and a further subgrouping of PD-L1 expression status into high (50–89%) and very high (≥90%) PD-L1 expression. Interestingly, patients with brain metastases, a cohort not represented in the KEYNOTE-024 study [21], demonstrated similar survival to patients without brain metastases. We consider that these were a highly selected group of patients from the overall population of patients with brain metastases eligible for pembrolizumab monotherapy. This is supported by the finding that although inflammatory status was associated with the presence of brain metastases, the majority were SIPS 1. This highlights a significant limitation of studies such as ours, with a lack of patients eligible for, but deemed unfit for, systemic therapy with pembrolizumab. This limits the ability to draw conclusions about SIPS’ role as a predictive biomarker with respect to treatment choice.

As has previously been reported, a very high PD-L1 expression (i.e., ≥90%) was associated with improved survival in our cohort [16,17]. Interestingly, as demonstrated by Shah et al., this association was only observed in patients with non-squamous histology, noting the small numbers of patients with squamous histology in our cohort (*n* = 76). Although SIPS was not associated with PD-L1 expression, confirming their independent prognostic value, we find that the combination of very high PD-L1 expression and low systemic inflammation predicts the most favourable survival outcomes in our cohort. Banna et al. also evaluated the role of biomarkers of systemic inflammation in patients with NSCLC expressing PD-L1 ≥ 50% treated with pembrolizumab [22]. A combination of neutrophil/lymphocyte ratio (NLR) < 5 and high PD-L1 expression ≥ 80% identified a group of patients in whom the 2-year OS rate was 81%. Future work is required to assess the prognostic significance of PD-L1 expression as a continuous scale and to understand how this relates to biomarkers of systemic inflammation and survival outcomes.

Another weakness of our study was the lack of other inflammatory indices investigated. A broad range of prognostic biomarkers of systemic inflammation have been proposed, with many, like SIPS, initially developed exclusively for NSCLC patients receiving ICIs. However, to date, the optimal biomarker of systemic inflammation in the patient group has not been well defined. The inflammatory burden index (IBI), advanced lung cancer inflammation index (ALI), and mGPS have been proposed as an optimal inflammatory biomarker of OS in heterogenous cohorts of patients with NSCLC with respect to stage and treatment modalities. The performance of SIPS in comparison to these inflammatory biomarkers has not yet been investigated. However, Stares et al. found albumin and NC to be superior to the neutrophil/lymphocyte ratio, which forms the backbone of ALI and IBI, white cell count, platelet/lymphocyte ratio (PLR), and the prognostic nutritional index in their original study of SIPS. Larger, more comprehensive studies are required to address this unmet need.

We also highlight the need to explore dynamic changes in SIPS or other biomarkers of systemic inflammation during the treatment journey. Most studies investigating the prognostic value of biomarkers of systemic inflammation utilize biomarkers recorded prior to treatment initiation. We have, although, previously identified that SIPS is prognostic at the time of progression on immunotherapy. In further work, we aim to explore how SIPS changes through treatment with respect to treatment outcomes including disease response and survival.

## 5. Conclusions

The results of this study validate the use of SIPS as a reliable prognostic factor in patients with NSCLC expressing PD-L1 ≥ 50% treated with first-line pembrolizumab. SIPS stratifies survival in a clinically meaningful timeframe, particularly identifying patients with the highest levels of inflammation who experience poor survival outcomes. The addition of other clinicopathological prognostic factors may provide more granular predictive power. This objective information may be used alongside routine clinical assessments to inform decisions about treatment and follow-up in this patient group.

## Figures and Tables

**Table 1:** Baseline clinical and demographic characteristics stratified by SIPS.

Characteristic	Total	SIPS 0 (*n* = 144)	SIPS 1 (*n* = 93)	SIPS 2 (*n* = 20)	*p*-Value
Age					
<65	85 (33%)	41 (9%)	39 (42%)	5 (25%)	0.021
65–74	104 (40%)	55 (38%)	37 (40%)	12 (60%)	
≥75	68 (26%)	48 (33%)	17 (18%)	3 (15%)	
Sex					
Female	124 (48%)	74 (51%)	41 (44%)	9 (45%)	0.522
Male	133 (52%)	70 (49%)	52 (56%)	11 (55%)	
ECOG PS					
0	36 (14%)	26 (18%)	10 (11%)	0 (0%)	0.029
1	191 (74%)	103 (72%)	73 (79%)	15 (75%)	
2	30 (12%)	15 (10%)	10 (11%)	5 (25%)	
Histology					
Squamous	76 (30%)	44 (31%)	20 (22%)	12 (60%)	0.147
Non-squamous	181 (70%)	100 (69%)	73 (79%)	8 (40%)	
PD-L1					
<90%	148 (58%)	84 (58%)	53 (57%)	11 (55%)	0.951
≥90%	109 (42%)	60 (42%)	40 (43%)	9 (45%)	
Brain Metastases					
Absent	225 (88%)	131 (91%)	75 (81%)	19 (95%)	0.036
Present	32 (12%)	13 (9%)	18 (19%)	1 (5%)	

**Table 2:** The relationship between prognostic factors and progression-free survival and overall survival in patients with non-small-cell lung cancer treated with first-line pembrolizumab monotherapy.

	Progression-Free Survival				Overall Survival			
	Univariate		Multivariate		Univariate		Multivariate	
	HR (95% CI)	*p*	HR (95% CI)	*p*	HR (95% CI)	*p*	HR (95% CI)	*p*
Age (≤64, 65–74, ≥75)	1.07 (0.90–1.27)	0.451			1.12 (0.93–1.35)	0.236		
Sex (Female, Male)	1.31 (0.99–1.73)	0.058			1.32 (0.98–1.76)	0.066		
Performance Status (0, 1, 2)	1.28 (0.98–1.67)	0.067			1.38 (1.04–1.83)	0.027		
Histological Subtype (squamous, non-squamous)	0.81 (0.60–1.10)	0.182			0.75 (0.55–1.02)	0.065		
PD-L1 (<90%, ≥90%)	0.68 (0.51–0.90)	0.008	0.67 (0.51–0.89)	0.006	0.66 (0.49–0.90)	0.008	0.64 (0.47–0.87)	0.004
Brain Metastases (absent, present)	0.96 (0.63–1.46)	0.845			1.02 (0.66–1.59)	0.919		
Neutrophils (≤7.5 × 10^9^/L, >7.5 × 10^9^/L)	1.60 (1.21–2.11)	0.001			1.74 (1.30–2.34)	<0.001		
Albumin (≥35 g/L, <35 g/L)	1.90 (1.22–3.00)	0.005			2.05 (1.30–3.24)	0.002		
Scottish Inflammatory Prognostic Score (0, 1, 2)	1.55 (1.25–1.92)	<0.001	1.56 (1.25–1.93)	<0.001	1.66 (1.33–2.09)	<0.001	1.66 (1.32–2.08)	<0.001

**Table 3:** The relationship between Scottish Inflammatory Prognostic Score (SIPS) and progression-free survival and overall survival at 3 months, 6 months, and 12 months in patients with non-small-cell lung cancer receiving first-line pembrolizumab monotherapy.

	SIPS	Patients (*n* (%))	Median (IQR) Months	*p*	Survival at 3 Months (*n* (%))	Survival at 6 Months (*n* (%))	Survival at 12 Months (*n* (%))
PFS	0	143 (56%)	14.0 (6.0–40.0)	<0.001	125 (87%)	110 (77%)	69 (48%)
	1	93 (36%)	7.9 (2.6–23.6)		67 (72%)	56 (60%)	36 (39%)
	2	20 (8%)	2.5 (1.6–7.6)		10 (50%)	8 (40%)	4 (20%)
OS	0	143 (56%)	23.4 (8.4–n/r)	<0.001	138 (97%)	124 (87%)	92 (64%)
	1	93 (36%)	16.4 (3.3–40.7)		71 (76%)	63 (68%)	49 (53%)
	2	20 (8%)	3.7 (2.2–9.7)		12 (60%)	9 (45%)	4 (20%)

n/r: not reached.

**Table 4:** The relationship between the Scottish Inflammatory Prognostic Score and PD-L1 expression status with respect to progression-free survival and overall survival patients with non-small-cell lung cancer receiving first-line pembrolizumab monotherapy.

			SIPS			*p*
			0	1	2	
PD-L1	<90%	PFS (Median (IQR))	*n* = 84 8.8 (5.5–28.7)	*n* = 107 6.6 (2.2–17.1)	*n* = 11 2.5 (1.2–7.6)	0.024
	≥90%		*n* = 60 18.9 (6.8–60.4)	*n* = 40 10.7 (4.5–30.4)	*n* = 9 2.5 (1.9–7.8)	0.003
*p*			0.011	0.182	0.790	
			**SIPS**			***p***
			**0**	**1**	**2**	
PD-L1	<90%	OS (Median (IQR))	*n* = 84 22.4 (7.9–42.6)	*n* = 107 10.0 (2.8–33.4)	*n* = 11 2.5 (1.8–10.7)	0.001
	≥90%		*n* = 60 28.2 (14.0–n/r)	*n* = 40 20.9 (10.3–55.5)	*n* = 9 6.3 (3.4–9.7)	0.004
*p*			0.041	0.055	0.329	

n/r: not reached.

## Data Availability

Research data are stored in an institutional repository and will be shared upon reasonable request to the corresponding author.

## References

[1] Sung H., Ferlay J., Siegel R.L., Laversanne M., Soerjomataram I., Jemal A., Bray F. (2021). Global Cancer Statistics 2020: GLOBOCAN Estimates of Incidence and Mortality Worldwide for 36 Cancers in 185 Countries. CA Cancer J. Clin..

[2] Reck M., Rodríguez–Abreu D., Robinson A.G., Hui R., Csőszi T., Fülöp A., Gottfried M., Peled N., Tafreshi A., Cuffe S. (2019). Updated Analysis of KEYNOTE-024: Pembrolizumab Versus Platinum-Based Chemotherapy for Advanced Non-Small-Cell Lung Cancer With PD-L1 Tumor Proportion Score of 50% or Greater. J. Clin. Oncol..

[3] De Castro G., Kudaba I., Wu Y.-L., Lopes G., Kowalski D.M., Turna H.Z., Caglevic C., Zhang L., Karaszewska B., Laktionov K.K. (2023). Five-Year Outcomes with Pembrolizumab Versus Chemotherapy as First-Line Therapy in Patients with Non–Small-Cell Lung Cancer and Programmed Death Ligand-1 Tumor Proportion Score ≥ 1% in the KEYNOTE-042 Study. J. Clin. Oncol..

[4] Gadgeel S., Rodríguez-Abreu D., Speranza G., Esteban E., Felip E., Dómine M., Hui R., Hochmair M.J., Clingan P., Powell S.F. (2020). Updated Analysis From KEYNOTE-189: Pembrolizumab or Placebo Plus Pemetrexed and Platinum for Previously Untreated Metastatic Nonsquamous Non–Small-Cell Lung Cancer. J. Clin. Oncol..

[5] Paz-Ares L., Vicente D., Tafreshi A., Robinson A., Parra H.S., Mazières J., Hermes B., Cicin I., Medgyasszay B., Rodríguez-Cid J. (2020). A Randomized, Placebo-Controlled Trial of Pembrolizumab Plus Chemotherapy in Patients with Metastatic Squamous NSCLC: Protocol-Specified Final Analysis of KEYNOTE-407. J. Thorac. Oncol..

[6] Hanahan D., Weinberg R.A. (2011). Hallmarks of Cancer: The Next Generation. Cell.

[7] Mezquita L., Auclin E., Charrier M., Ferrara R., Masip J.R., Planchard D., Aix S.P., Paz-Ares L., Lahmar J., Leroy L. (2017). The Lung Immune Prognostic Index (LIPI), a predictive score for immune checkpoint inhibitors in advanced non-small cell lung cancer (NSCLC) patients. Ann. Oncol..

[8] McMillan D.C. (2013). The systemic inflammation-based Glasgow Prognostic Score: A decade of experience in patients with cancer. Cancer Treat. Rev..

[9] Benitez J.C., Recondo G., Rassy E., Mezquita L. (2020). The LIPI score and inflammatory biomarkers for selection of patients with solid tumors treated with checkpoint inhibitors. Q. J. Nucl. Med. Mol. Imaging.

[10] Mezquita L., Arbour K., Auclin E., Saravia D., Rizvi H., Hendriks L., Planchard D., Park W., Nadal E., Rodriguez J.R. (2018). Derived neutrophil-to lymphocyte ratio (dNLR) change between baseline and cycle 2 is correlated with benefit during immune checkpoint inhibitors (ICI) in advanced non-small cell lung cancer (NSCLC) patients. Ann. Oncol..

[11] Russo A., Franchina T., Ricciardi G.R., Battaglia A., Scimone A., Berenato R., Giordano A., Adamo V. (2018). Baseline neutrophilia, derived neutrophil-to-lymphocyte ratio (dNLR), platelet-to-lymphocyte ratio (PLR), and outcome in non small cell lung cancer (NSCLC) treated with Nivolumab or Docetaxel. J. Cell Physiol..

[12] Banna G., Cortellini A., Cortinovis D., Tiseo M., Aerts J., Barbieri F., Giusti R., Bria E., Grossi F., Pizzutilo P. (2021). The lung immuno-oncology prognostic score (LIPS-3): A prognostic classification of patients receiving first-line pembrolizumab for PD-L1 ≥ 50% advanced non-small-cell lung cancer. ESMO Open.

[13] Ramspek C.L., Jager K.J., Dekker F.W., Zoccali C., van Diepen M. (2021). External validation of prognostic models: What, why, how, when and where?. Clin. Kidney J..

[14] Stares M., Ding T., Stratton C., Thomson F., Baxter M., Cagney H., Cumming K., Swan A., Ross F., Barrie C. (2022). Biomarkers of systemic inflammation predict survival with first-line immune checkpoint inhibitors in non-small-cell lung cancer. ESMO Open.

[15] Ricciuti B., Elkrief A., Alessi J.V.M., Wang X., Barrichello A.P.d.C., Pecci F., Lamberti G., Lindsay J., Sharma B., Felt K. (2022). Three-year outcomes and correlative analyses in patients with non–small cell lung cancer (NSCLC) and a very high PD-L1 tumor proportion score (TPS) ≥ 90% treated with first-line pembrolizumab. J. Clin. Oncol..

[16] Shah M., Marmarelis M.E., Mamtani R., Hennessy S. (2022). Association Between Survival and Very High Versus High PD-L1 Expression in Patients Receiving Pembrolizumab as First-line Treatment for Advanced Non-Small Cell Lung Cancer. Clin. Lung Cancer.

[17] Aguilar E., Ricciuti B., Gainor J., Kehl K., Kravets S., Dahlberg S., Nishino M., Sholl L., Adeni A., Subegdjo S. (2019). Outcomes to first-line pembrolizumab in patients with non-small-cell lung cancer and very high PD-L1 expression. Ann. Oncol. Off. J. Eur. Soc. Med. Oncol..

[18] Navani N., Baldwin D.R., Edwards J.G., Evison M., McDonald F., Nicholson A.G., Fenemore J., Sage E.K., Popat S. (2022). Lung Cancer in the United Kingdom. J. Thorac. Oncol..

[19] Brown K.F., Rumgay H., Dunlop C., Ryan M., Quartly F., Cox A., Deas A., Elliss-Brookes L., Gavin A., Hounsome L. (2018). The fraction of cancer attributable to modifiable risk factors in England, Wales, Scotland, Northern Ireland, and the United Kingdom in 2015. Br. J. Cancer.

[20] Dolan R.D., Daly L.E., Simmons C.P., Ryan A.M., Sim W.M., Fallon M., Power D.G., Wilcock A., Maddocks M., Bennett M.I. (2020). The Relationship between ECOG-PS, mGPS, BMI/WL Grade and Body Composition and Physical Function in Patients with Advanced Cancer. Cancers.

[21] Reck M., Rodríguez-Abreu D., Robinson A.G., Hui R., Csőszi T., Fülöp A., Gottfried M., Peled N., Tafreshi A., Cuffe S. (2016). Pembrolizumab versus Chemotherapy for PD-L1-Positive Non-Small-Cell Lung Cancer. N. Engl. J. Med..

[22] Banna G.L., Signorelli D., Metro G., Galetta D., De Toma A., Cantale O., Banini M., Friedlaender A., Pizzutillo P., Garassino M.C. (2020). Neutrophil-to-lymphocyte ratio in combination with PD-L1 or lactate dehydrogenase as biomarkers for high PD-L1 non-small cell lung cancer treated with first-line pembrolizumab. Transl. Lung Cancer Res..

